# Using massively parallel shotgun sequencing of maternal plasmatic cell-free DNA for cytomegalovirus DNA detection during pregnancy: a proof of concept study

**DOI:** 10.1038/s41598-018-22414-6

**Published:** 2018-03-12

**Authors:** Virginie Chesnais, Alban Ott, Emmanuel Chaplais, Samuel Gabillard, Diego Pallares, Christelle Vauloup-Fellous, Alexandra Benachi, Jean-Marc Costa, Eric Ginoux

**Affiliations:** 1 Life&Soft, Plessis-Robinson, France; 2 AP-HP, Hôpital Paul Brousse, Groupe Hospitalier Universitaire Paris-Sud, Virologie, Université Paris-Sud, INSERM U1193 Villejuif, France; 3 AP-HP, Hôpital Antoine Béclère, Service de Gynécologie-Obstétrique et Médecine de la Reproduction, Université Paris-Sud, Clamart, France; 4 Laboratoire CERBA, Saint-Ouen-l’Aumône, France

## Abstract

Human cytomegalovirus (HCMV) primary infections of pregnant women can lead to congenital infections of the fetus that could have severe impacts on the health of the newborn. Recent studies have shown that 10–100 billion DNA fragments per milliliter of plasma are circulating cell-free. The study of this DNA has rapidly expanding applications to non-invasive prenatal testing (NIPT). In this study, we have shown that we can detect viral specific reads in the massively parallel shotgun sequencing (MPSS) NIPT data. We have also observed a strong correlation between the viral load of calibration samples and the number of reads aligned on the reference genome. Based on these observations we have constructed a statistical model able to quantify the viral load of patient samples. We propose to use this new method to detect and quantify circulating DNA virus like HCMV during pregnancy using the same sequencing results as NIPT data. This method could be used to improve the NIPT diagnosis.

## Introduction

The use of fetal cell-free DNA (cfDNA) for aneuploidy screening has spread rapidly since 2011 in clinical practices. Current approaches involve massively parallel shotgun sequencing (MPSS), targeted sequencing and assessment of single nucleotide polymorphism (SNP) differences between mother and fetus^1–3^. At the moment, MPSS approaches for the detection of fetal aneuploidies are more extensively validated than targeted approaches with regard to sensitivity and specificity^4^. Furthermore, MPSS offers the possibility to explore much more than the sole fetal and maternal genome. For example, it could be extended to the discovery or analysis of non-human cfDNA such as viruses.

Human cytomegalovirus (HCMV) is a DNA β-herpesvirus of critical importance to human health during pregnancy^5^. HCMV maternal primary infection during pregnancy could lead to a congenital infection and could have severe clinical complications for the newborn: sensorineural hearing loss (SNHL), visual impairment, motor and cognitive defects or transient symptoms like hepatosplenomegaly, thrombocytopenia and jaundice^6^. The overall prevalence of congenital HCMV infection has been estimated to be 0.7%^7^ with the highest transmission rate occurring after primary infection of immunonegative women. However, HCMV reactivation or reinfection in immunized women may also lead to fetal transmission of the virus, but the transmission rate is unknown. The vertical transmission of virus from the infected mother to the fetus via the uterine-placental interface occurs in 40 to 50% of primary infection cases^8,9^. Interestingly, HCMV infection is a risk factor for fetal intrauterine growth restriction (IUGR), even in the absence of virus transmission to the fetus, suggesting that HCMV infection of the placenta can lead to dysfunctions in nutrient and oxygen delivery^10^. About 12% of infected newborns will have specific symptoms at birth and 40 to 58% of them will have permanent sequelae. Furthermore, 13% of newborns without symptoms at birth will develop permanent sequelae during their first year of life, mainly hearing impairment^7^.

The risk of transmission increases with the progression of the pregnancy^11^. A lower transmission rate is observed when the maternal infection occurs in the first trimester (before 20 weeks of gestation). On the other hand, the impact of maternal infection on the fetus’ health seems to be higher when the maternal infection occurs at the beginning of pregnancy (before 20 weeks of gestation)^12^. Because the HCMV viral load increases regularly during the active phase of infection, it can be used to estimate the beginning of the infection^13,14^.

The HCMV infection is mostly asymptomatic in immunocompetent persons and can be unnoticed in women during their pregnancy. Although screening policies may be different depending on the country and are currently still debated, a serologic screening at the beginning of pregnancy may be used to attest the immune status of pregnant women. For seronegative women, a follow-up can be performed during pregnancy to detect a seroconversion^15^. HCMV specific immunoglobulin M (IgM) antibodies can possibly indicate an acute or a recent infection but can also be due to other causes (i.e.: long-term persisting IgM, cross-reaction, secondary HCMV infection or nonspecific stimulation of the immune system). Consequently, diagnosis of a primary infection cannot only rely on a positive IgM test result and must be confirmed by performing immunoglobulin G (IgG) avidity. For now, diagnosis of HCMV primary infection during pregnancy is considered reliable but diagnosis of HCMV non-primary infection is much more questionable and currently not routinely performed during pregnancy^16,17^. Finally, ultrasound fetal abnormalities can indicate a congenital infection and could be detected with a suitable follow-up of women at risk of seroconversion during their pregnancy. But, if ultrasound is one of the best markers of congenital HCMV infection, the signs are not specific and are not always present^18,19^.

New approaches to diagnose viral infection with the help of NGS are emerging^20^. The purpose of this study is to evaluate a method to detect and quantify circulating viral DNA during pregnancy in maternal plasma, by using the same sequencing data from the noninvasive prenatal whole genome sequencing data. This method uses the human unmappable reads to search for viral specific reads and quantify the absolute viral load.

## Results

### Viral DNA does not interfere with human DNA sequencing

**Figure 1:**
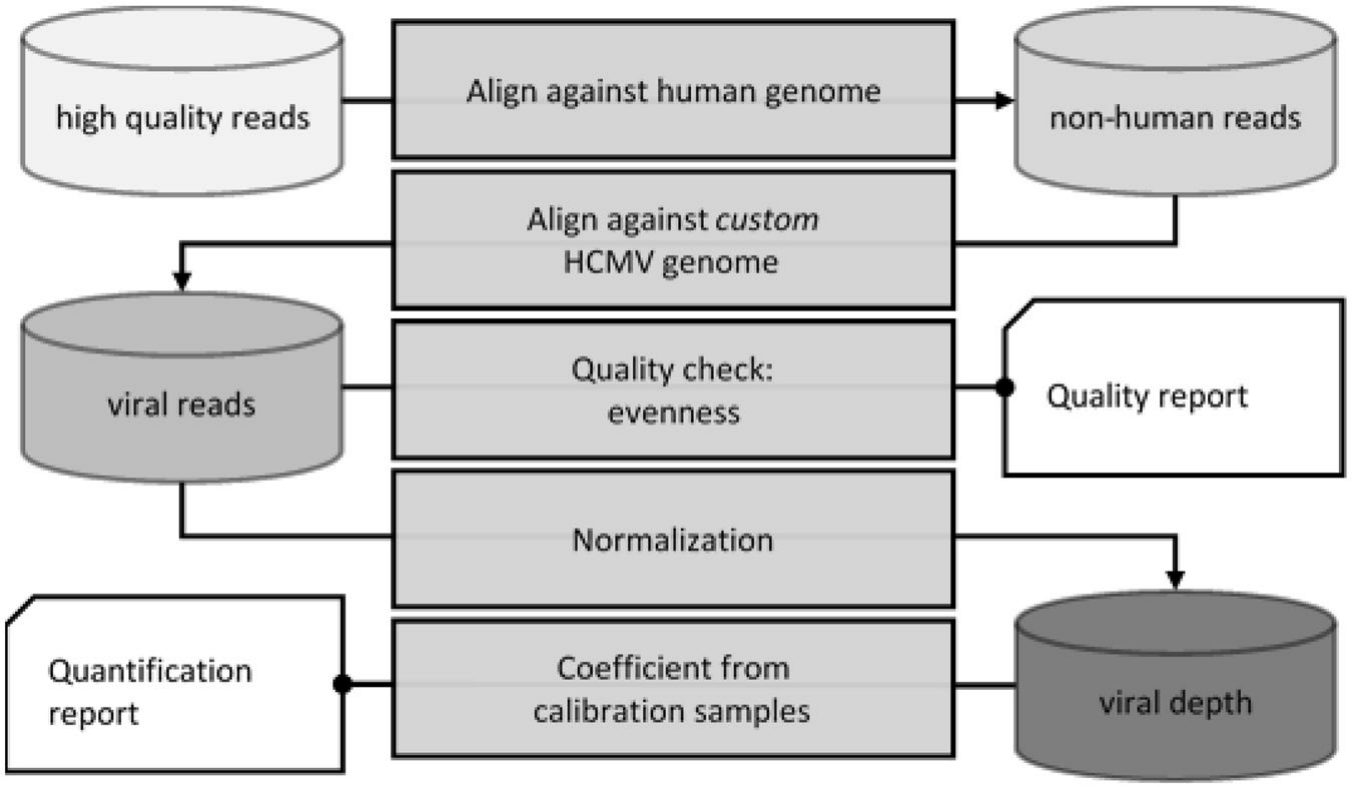
Description of the analysis pipeline. Plasma samples were collected and whole genome sequencing of cfDNA was performed. For each sample, human reads were filtered to keep only exogenous reads containing viral reads. The estimation of viral load is performed after the quantification of reads specifically aligned with high quality on viral genome.

We sequenced 27 calibration HCMV samples and 58 negative controls, and analyzed results as described in Fig. 1. An average of 18 million reads (range: 11–28) were obtained per sample after MPSS. A median of 14.1% of the reads (range: 4.3–18.5) were considered as bad quality reads and removed from the analysis. A median of 78.4% high-quality reads (range: 74,7–82,2%) were mapped on human genome (GRCh38) and filtered for further analysis (Supplemental Fig. 1A). The alignment parameters at this step don’t allow mismatches thus we are confident to discard only reads from the human genome. We observed no difference in the number of sequenced reads between positive and negative calibration samples (Supplemental Fig. 1B). After alignment to the human genome (GRCh38), we observed an average of 14 million reads (range: 7–22) of human mapped reads. The proportion of reads aligned to the human genome was the same between positive and negative samples (Supplemental Fig. 1C). These observations suggest that the presence of viral DNA in a biologic sample does not impact the total number or the quality of reads obtained after MPSS.

Finally, for all calibration samples, a median of 19.5% (range: 17.3–20.5) was considered as unknown origin reads and could gather artefactual reads with low complexity or repeated sequences that cannot be aligned on human genome or reads with exogenous origins like microorganism reads (Supplemental Fig. 1A).

### Non-specific alignment on the HCMV published reference genome

**Figure 2:**
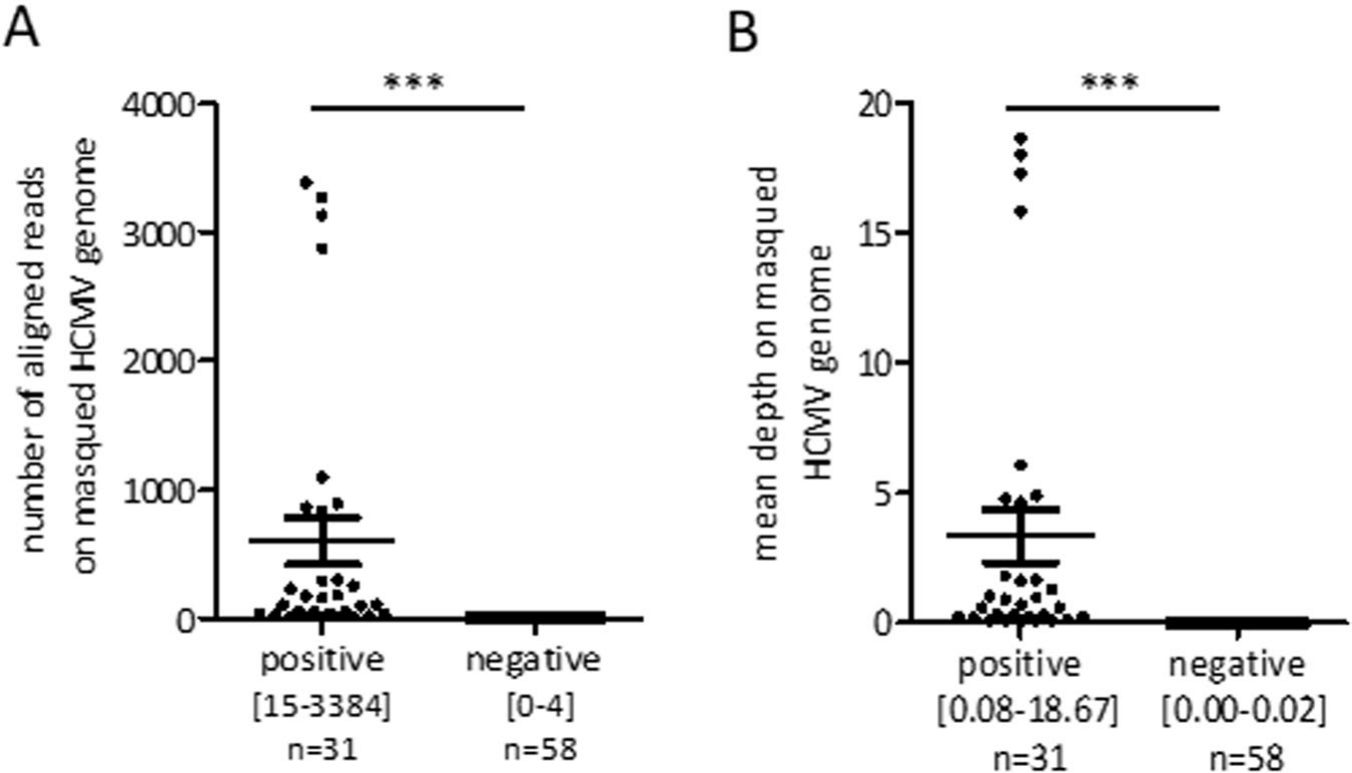
Analysis of the presence of HCMV DNA in calibration samples. (**A**) Representation of the number of reads mapped to the masked HCMV reference for positive calibration samples (with viral load between 10 copies/mL and 2500 copies/mL) and negative controls. The mean number of reads ± standard error of the mean (SEM) is represented for both groups. The range values of read numbers [min-max] and sample number in each group are also reported. ***Represents a p-value < 0.001 (Mann–Whitney test). (**B**) Representation of the mean depth of positive calibration samples (with a viral load between 10 copies/mL and 2500 copies/mL) and negative controls to the masked HCMV reference. The mean depth ± standard error of the mean (SEM) is represented for both groups. The range values of read numbers [min-max] and sample numbers in each group are also reported. ***Represents a p-value < 0.001 (Mann–Whitney test).

We aligned all non-human reads to the HCMV reference NC_006273, and thus retrieved HCMV reads. For negative samples, we observed a median of 7.00 reads mapped to the HCMV genome (range: 2.00–15.50) after normalization, as described in the methods section (Supplemental Fig. 2A). We then computed the normalized standard deviation of depth on each genome position for all negative controls and noticed a large standard deviation that reflects a non-specific alignment of reads at some genome locations (Supplemental Fig. 2B). Indeed, when we looked at the alignment files, we noticed that mapped reads seem to accumulate on a limited number of regions on the HCMV genome. These bins appear to be the same for all studied negative samples and fit with low complexity sequences. To reduce these biases, we masked these regions in the HCMV genome. We then realigned negative samples to the HCMV masked genome and found a significant decrease of non-specific alignments with a median of 0.00 mapped reads (range: 0.00–4.00) (p-value < 0.001, Wilcoxon signed rank test for paired samples). Finally, we checked the validity of our new reference genome by aligning positive HCMV samples on HCMV masked genome, and we observed a median of 111 mapped reads significantly different from the negative samples. (range: 15–3384, p-value < 0.001 Mann-Whitney test) (Fig. 2A).

The absence of aligned reads to the HCMV masked genome only for negative HCMV samples indicates a specific alignment only for samples with a positive viremia. The comparison of the normalized standard deviation of depth for negative controls with non-modified and masked HCMV reference was used to determine a threshold indicating a homogeneous alignment throughout the genome (Supplemental Fig. 2C and D).

### Correlation between depth and viral load

As expected, depth on the HCMV genome in positive calibration samples is significantly different from the negative controls (3.36 (range: 0.08–18.67) vs 0.00 (range: 0.00–0.02), p-value < 0.001 Mann-Whitney test). Interestingly, we observed no overlap between the two groups (Fig. 2B). A receiver operating characteristic (ROC) analysis was performed to assess the sensitivity and specificity of the method in differentiating samples based on the presence of HCMV circulating DNA. It allowed us to determine a mean depth value threshold to classify samples with a positive and a negative viremia for HCMV with a sensitivity of 100% [88–100%] and a specificity of 100% [94–100%].

**Figure 3:**
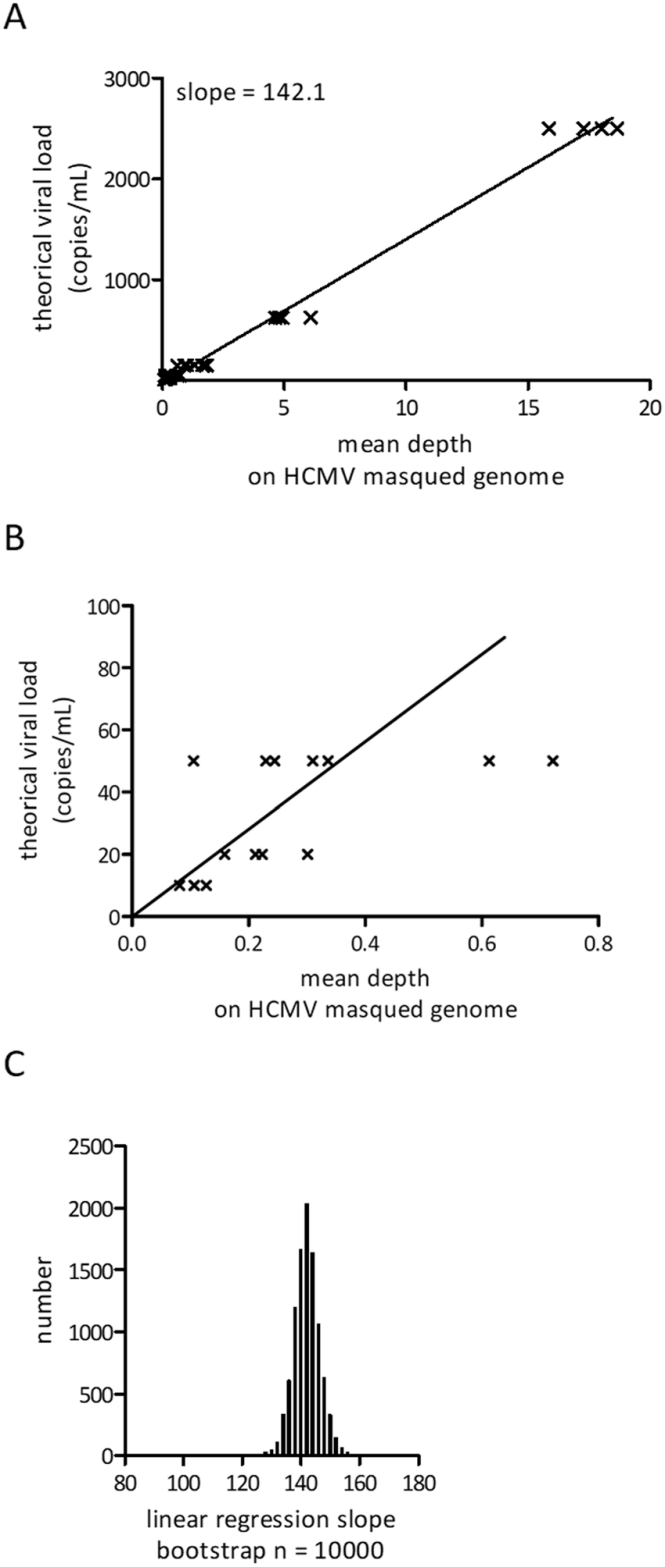
Correlation between depth and theoretical viral load of HCMV calibration samples. (**A**) Correlation between the mean depth obtained from all HCMV calibration samples with known viral loads. The linear regression curve is also shown. (**B**) Same data as A but focused on low calibration samples. (**C**) Repartition of all linear regression slopes obtained after bootstrap tests (n = 10000) based on all HCMV calibration samples.

We also observed a positive correlation between the number of reads aligned to the HCMV genome and the theoretical viral load of calibration samples (Supplemental Fig. 3). A linear regression model enabled the identification of a coefficient that allows the estimation of the viral load for unknown samples (Fig. 3A) (R² = 0.99, p-value < 0.001). We checked and validated our model on the lowest concentration titration samples (Fig. 3B). To improve this model, we performed a bootstrapping. A random sampling with replacement of calibration samples was performed 10000 times. After each sampling, the linear regression analysis was performed, and values were then plotted and compared to the initial coefficient (Fig. 3C). Coefficients ranged from 98 to 158 (Supplemental Fig. 4), with a mean value equal to 141.9 which was close to the initial coefficient 142.1. Using a bootstrap procedure, a 99% viral load confidence interval was assigned for all testing samples. Corresponding coefficients are 134.7 and 149.7.

The computed viral load is normalized with sequencing depth. We consider that our detection limit is 10 copies/mL because we don’t have any calibration sample below. However, this detection limit depends a lot on sequencing depth, and raises a new question: What is the minimal sequencing depth to obtain reliable detection of infection? To answer this question, we performed rarefaction analysis by sub-sampling reads from two calibration samples with viral load of 2500 copies/ml and 10 copies/ml, to simulate samples with lower depth. For both studied calibration samples, we observed an increase in the variation coefficient of the estimated viral load when the sequencing depth decreases. For sample with highest viral load, we determined as critical the threshold of 100000 reads. Below this threshold the variation coefficient significantly increases, and the sensitivity of the quantification decreases. The only false negative results appear at a simulated sample depth of 25000 reads. Whatever, for 17 out of 20 draws, the estimated viral loads are higher than 1400 copies/ml. For the lowest viral load, the variation coefficient starts to increase from 5 million reads with 1 out of 20 false negative results detected at this depth (Supplemental Figure 5). These results were confirmed with two other calibration samples sequenced in a different series. The false negative samples raise up when the expected number of reads is so low that sometimes we obtain no read at all, then the sample is automatically negative.

We conclude that a sequencing with 5 million reads or less should not be considered for viral load quantification. From this depth, there is a to high rate of false negative and the absolute quantification is not precise enough to be used as a diagnosis test.

### Specific alignment on HCMV masked genome

To confirm the specificity of the alignment, we mapped reads from HCMV calibration samples to different DNA viral references: all herpesvirus families (HSV), hepatitis B (HBV), parvovirus B19 and 60 papillomavirus strains. The genome length of these DNA viruses ranged from 3 kb to 235 kb. To be able to compare the results between different species we considered the depth coverage as described in the methods section.

For HCMV positive samples we observed specific alignments to the HCMV reference genome, within a depth range from 7.23E-05 to 71.25E-02 for all viral concentrations. We observed very few non-specific alignments against other HSV reference genomes (Table 1). For all samples, these alignments are equivalent between positive and negative calibration samples.

**Table 1:** Median of DNA virus reference genome depth for HCMV callibration and other control samples.

Samples	Median of genome depth									
	HBV	HHV-1	HHV-2	HHV-3	HHV-4	HHV-5	HHV-6A	HHV-6B	HHV-7	HHV-8
HCMV2500	0	1,97E-05	4,55E-05	0	0	1,25E-02	6,92E-05	0	3,92E-05	4,38E-05
HCMV 625	0	1,97E-05	8,44E-05	0	5,81E-06	3,82E-03	4,40E-05	<1E-06	6,54E-05	3,65E-05
HCMV 150	0	1,32E-05	5,19E-05	<1E-06	0	8,85E-04	<1E-06	3,09E-05	5,23E-05	4,38E-05
HCMV 50	0	1,32E-05	4,55E-05	<1E-06	5,81E-06	2,21E-04	2,52E-05	1,23E-05	3,27E-05	4,38E-05
HCMV 20	0	1,32E-05	4,55E-05	<1E-06	5,81E-06	1,32E-04	0	0	4,58E-05	5,11E-05
HCMV 10	0	1,32E-05	3,90E-05	<1E-06	0	7,23E-05	0	0	2,61E-05	2,19E-05
HCMV 0	0	<1E-06	5,84E-05	<1E-06	0	0	0	0	3,27E-05	2,19E-05
HBV control	5,66E + 01	0	0	0	0	0	0	0	0	0
EBV control	0	<1E-06	<1E-06	0	2,95E-02	<1E-06	3,14E-05	0	3,27E-05	2,92E-05

An alignment of all HCMV callibration samples was performed against all herpesvirus family species (HSV-1, HSV-2, HSV-3, HSV-4 or EBV, HSV-5 or HCMV, HSV-6A, HHV-6B, HSV-7 and HSV-8) and hepatitis B (HBV). Two control samples positive for EBV and HBV samples were also performed against these references.

To confirm the alignment specificity, we studied two other control samples positive for hepatitis B (HBV) and Epstein-Barr virus (EBV or HSV-4) and respectively computed a 5.66E01 depth coverage of the HBV genome and 2.95E-02 depth coverage of the EBV genome. Once again, we did not observe non-specific alignments against other reference genomes (Table 1).

These observations confirm the specific alignment on HCMV of 27 bp reads after MPSS of cell free plasmatic DNA samples.

### Identification of positive HCMV samples with a low viral load

**Figure 4:**
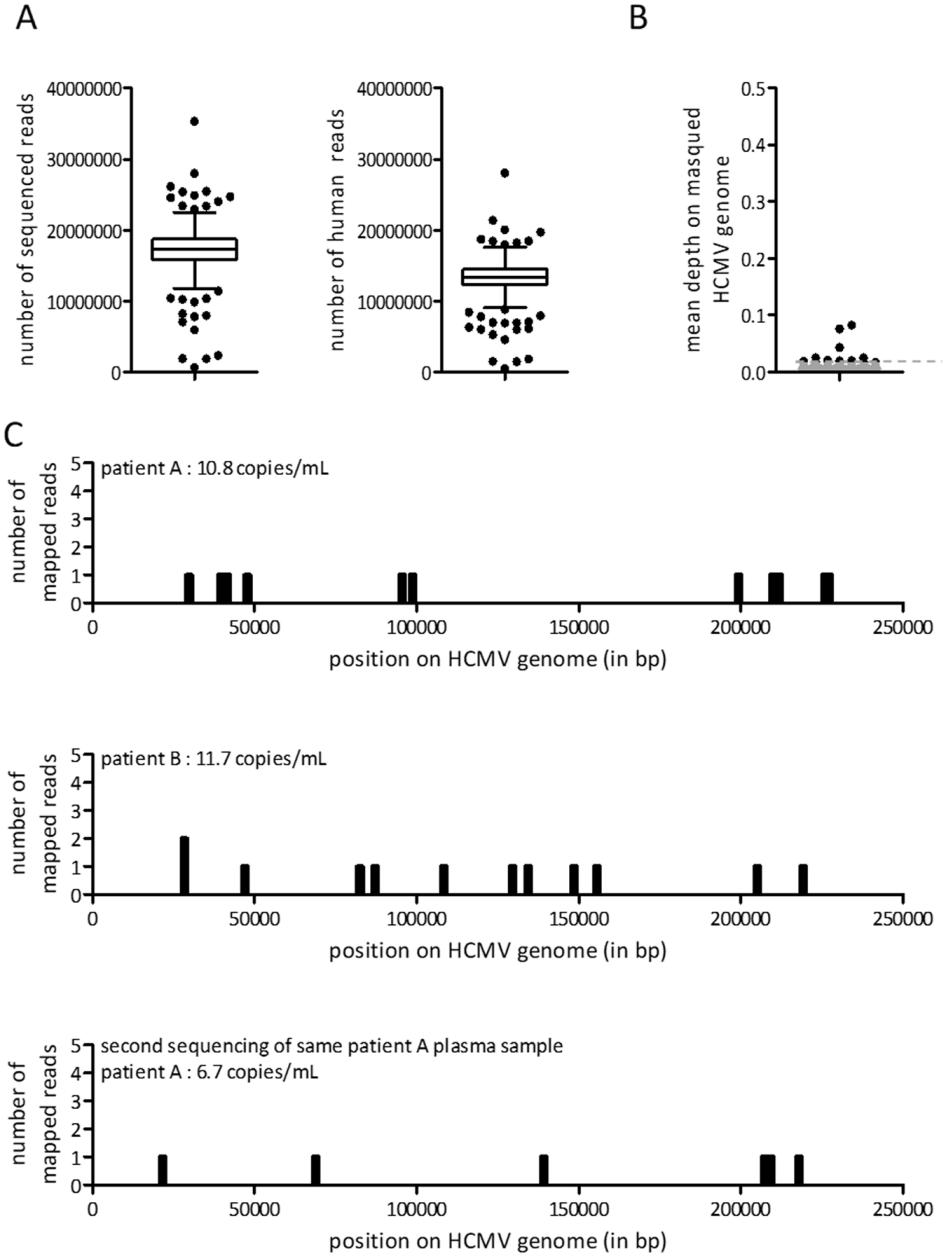
HCMV sequencing results of the retrospective pregnant patient cohort. (**A**) Summary of raw sequencing results of the 538 plasma samples. The total number of sequenced reads and the total number of human sequenced reads were represented. Data are shown as Tukey boxplots. The bottom and top of the box indicates the upper and lower quartiles and the band inside the box indicates the median. (**B**) Mean depth on masked HCMV samples for the 538 plasma samples were represented. The grey zone represents samples with a mean depth lower than the positive threshold for detection of HCMV circulating DNA. (**C**) Detailed alignment of reads for positive samples. For each plot, the number of reads aligned at all HCMV genome positions (in bp) is represented.

We studied a cohort of 538 pregnant women, corresponding to 574 plasma samples, to identify HCMV viremia positive samples. For 36 women, plasma was sequenced two times from the same sample or from two different samples with a maximal span of two months. The sequencing of these samples has been performed in the same conditions as the calibration samples. A mean number of 17 million reads per sample was obtained with a minimum of 5.9 million reads and a maximum of 35.3 million reads sequenced. An average of 77% of reads were correctly aligned to the human genome and discarded for the rest of the analysis (Fig. 4A).

After sample alignment to the HCMV genome, we computed the viral depth for each sample. For two samples, a depth superior to the previously determined threshold was observed, highlighting the presence of circulating HCMV DNA (Fig. 4B). For those two samples, we sequenced 15.9 million and 16.1 million reads and counted respectively 11 and 12 reads regularly mapped to the HCMV genome (Fig. 4C). When we estimated the viral load of the two samples we found that the two positive samples had a viral load equal to 10.8 (CI99% ± 0.57) and 11.7 (CI99% ± 0.62) copies/mL. We had biological material to resequence one of these samples (patient A) and we found again a significant number of reads mapped to the HCMV genome (Fig. 4C).

For 357 out of 538 samples, HCMV IgG dosage was performed to test the immune status of women at the beginning of their pregnancy. 204 women were immunized against HCMV based on the presence of HCMV IgG. The serologic status was known for one of the two identified patients. The presence of HCMV IgG and IgM for patient A suggests an immunity against HCMV so that the presence of HCMV cell free DNA in this patient could be explained either by an acute infection or by the release of intra-cellular viruses. To validate the specificity of these results, we mapped reads from these two positive samples against all other herpesvirus reference genomes. We did not observe other viral alignment, confirming the specific positive viremia for HCMV for these two patients.

### Application to other viral DNA with impact on pregnancy

Other DNA viruses can have a clinical impact during pregnancy on both mother and fetus. For 369 patients of our cohort, immunologic tests were performed to detect infected women for HBV virus. The serology was positive for three samples. When we mapped all these samples’ reads against the HBV reference genome, we found two samples with a mean depth higher than the positive threshold, suggesting the presence of circulating HBV DNA (Supplemental Figure 6A) in the plasma of these samples. We observed the alignment of respectively two and one reads on HBV reference for patient A and patient C. Interestingly, patient A has already been identified by our method as positive for HCMV viremia. The resequencing of patient A’s plasma confirmed the presence of circulating HBV DNA for this patient (Supplemental Figure 6B). These two patients correspond to two of the three patients of the cohort with a positive HBV serology identified by immunological testing. No reads were found to be aligned on HBV genome for the last patient with a positive HBV serology.

Finally, we saw with these additional results that our tool can detect other positive viremia than HCMV that are in tune with the serologic status. Our tool did not find false positive results in the 369 studied patients.

## Discussion

With 10–100 billion fragments per milliliter of plasma, cfDNA is an information-rich window into human physiology, with rapidly expanding applications in several domains. It is classically used in a clinical setting to screen for fetal aneuploidies and other subchromosomal abnormalities during pregnancy^1^ or to detect and monitor cancer on liquid biopsy^21–23^. Shotgun sequencing has also been used for several years for microbiome samples study, like the human plasmatic microbiome characterization, and has become a standard tool in pathogen discovery. Different tools have been developed to detect different microorganisms in a single biological sample like viruses, bacteria or fungi, and to estimate their relative abundance. These methods are not targeted and do not allow the absolute quantification of microorganisms^24–26^. The MPSS can also be used for more targeted analysis to detect the presence of specific microorganisms with clinical impact on human health. For example, it can be used to detect the presence of circulating HCMV sequences after lung transplantation to monitor the graft reject^27^.

In this study, we used maternal plasmatic cfDNA to improve information that can be useful to predict the health status of the fetus during pregnancy. We used an approach based on NIPT sequencing results to determine the maternal viral infection status. We have demonstrated a linear correlation between the theoretical viral load of calibration samples and the mapping depth on the HCMV reference genome. Based on this observation, we have constructed a statistical model to determine viral concentrations from any patient’s sample with unknown viral load.

Our method was validated on a cohort of 538 pregnant women. In this validation cohort, we found two positive samples for HCMV with a very low viral load. One of these samples was immunopositive for HCMV, suggesting that the small amount of viral DNA found in the maternal plasma could be the consequence of a viral reactivation or HCMV reinfection or could originate from few blood that have previously integrated the virus.

Even though the HCMV infection is unsafe for the fetus, it is symptomless for most people including pregnant women. Regarding the ability of the current HCMV detection methods, our developed model seems to be an interesting approach. With a very high sensitivity, this sequencing-based detection method could make HCMV infection evident during pregnancy, even with a plasmatic viral load as low as 10 copies/mL. NIPT is performed on maternal plasma samples at the end of the first trimester of pregnancy, a critical period for maternal HCMV infection that is predicted to have the higher impact on fetus health^12^. The suspicion of a maternal infection early in pregnancy is needed by clinicians to engage a more specific follow-up. For example, treatments can be proposed to decrease the risk of HCMV congenital infection. It has been suggested that hyperimmune globulin can prevent the rate of intrauterine transmission of HCMV during pregnancy^28^. Other approaches have also been developed to eliminate HCMV infected cells and could help to prevent congenital infection by blocking the virus expansion in maternal cells^29^.

Whatever, the viral load by itself is not predictive of an affected fetus, it would be interesting to identify more predictive markers of HCMV symptomatic congenital infection such as the genotype of the virus that has been suggested to be predictive of the presence and the gravity of sequelae in newborns^30,31^. Other markers like the total amount of cfDNA could indicate a placenta inflammatory response causing apoptosis and could act as an indicator of fetal distress^32^.

Lots of other DNA viruses can have a clinical impact during pregnancy on both mother and fetus^33^. In the same validation cohort, we found two positive samples for HBV that are also positive for the presence of the HBV antigen, confirming that our pipeline can detect different viral species. In the cohort, one patient has a positive HBV antigenemia but was negative for DNAemia by our analysis method. It could be the consequence of a non-replicative HBV infection resulting in a low level of circulating HBV DNA in the plasma of the pregnant woman or a lack of sensitivity of the method. Indeed, HBV detection by MPSS is more challenging than HCMV, whom genome is 78 times longer than the HBV genome which results in a lower probability of reads with HBV origin than with HCMV origin for the same viral load. Even if 5 million reads are enough for HCMV detection at the lowest viral load with the detection of a mean of 27 reads per cell free DNA samples, it is necessary to have a much higher sequencing depth to detect shorter DNA viral species like HBV at low viral load with the same number of reads detected.

With this study we demonstrate the feasibility to perform virus screening on sequenced maternal plasma. This sequencing can be proposed at the end of the first trimester of pregnancy. Indeed NIPT can become an affordable option in genetic prenatal care, for the detection of fetal karyotypic abnormalities but also for the detection of subchromosomal abnormalities^34,35^ and monogenic diseases^36,37^. With expected decrease in sequencing costs, MPSS seems to become the best approach. This study underlines the potential benefit of MPSS based monitoring of pregnant women to perform large prenatal screening based on a single test by the detection of both genetic abnormalities and maternal infections that could have a critical influence on fetus health. Our virus quantification algorithm has the potential to become an interesting complementary tool for non-invasive prenatal diagnosis because: (i) it is able to determine a plasmatic viral load with a 10 copies/mL threshold and can be performed on the same sequencing data that are generated for classical NIPT which are generally low depth sequencing results to reduce costs, (ii) the initial calibration of the method allows a reproducibility between results that is essential for its use as a standard test by different laboratories and (iii) it can be used to detect and quantify multiple viruses from a single DNA sequencing without modification of the experimental protocol. Furthermore, because serological tests can be difficult to interpret cases like secondary infection or viral reactivation, our approach can be an interesting complementary test to conclude on whether viral DNA is circulating in the maternal plasma. However, the aim of our study is not to overcome serology or any other existing test, but it could be an opportunity to raise an alert on pregnancy at risk. In case of a suspected maternal infection, the clinician should be able to provide an appropriate follow-up for the rest of the pregnancy by confirming the maternal infection and establishing the risk for the fetus to be also infected.

## Methods

### HCMV calibration samples

To mimic infected maternal plasma samples, we mixed plasma samples of non-infected women and plasma infected samples previously quantified by qPCR to make HCMV calibration samples of known viral loads. The qPCR was performed with the COBAS® AmpliPrep/COBAS® TaqMan® CMV Test kits from Roche Diagnostics Meylan, France. The claimed detection limit is 61 copies/mL and the quantification threshold is 150 copies/mL. The sequencing of calibration samples was performed in duplicate for each sample and over different sequencing runs. We studied 27 positive samples of 2500 copies/mL, 625 copies/mL, 150 copies/mL, 50 copies/mL, 20 copies/mL and 10 copies/mL in three or four distinct series. We also studied 58 negative plasma samples from noninfected women (Supplemental Table 1). The rarefaction analysis was performed by randomly choosing 20 subsections from the reads of a single fastq file to simulate samples with lower sequencing depth. For each calibration sample, we created 12 rarefaction levels to simulate samples with 15 million, 10 million, 5 million, 2 million, 1 million, 750000, 500000, 250000, 100000, 75000, 50000 and 25000 reads. For each rarefaction level, we determined the variance of the computed viral load and false negative rate (FNR). FNR is defined as number of positive samples that are below the detection threshold and so labeled as negative.

### Patients

All 538 patients were included in the DEPOSA study, for which the aim was to evaluate cfDNA trisomy 21, 18 and 13 screening using massive parallel sequencing as a primary screening. From May 2015 to February 2016 pregnant women undergoing first-trimester aneuploidy screening in nine French centers were enrolled. The institutional review board approved the study (CPP _Comités de protection des personnes_ N° 14–054) and a written informed consent was obtained from all patients. All methods were performed in accordance with the relevant guidelines and regulations defined by the institutional review board. Criteria for eligibility were to be at least 18 years of age and singleton pregnancy between 10 and 13 weeks of amenorrhea at the time of the blood collection. Gestational age was determined by the crown-rump length (CRL) measurement performed during ultrasound.

### Maternal plasmatic cfDNA massive parallel shotgun sequencing

The cfDNA was obtained by massive parallel shotgun sequencing approach, as described in the Jensen *et al*. study, including some slight modifications^1,38^. Maternal blood was collected in two cfDNA BCT Streck® tubes (10 mL each) and transported in a +4 °C environment to the clinical laboratory, where plasma was isolated within 4 days of collection by a double centrifugation procedure. Samples were rejected when the tubes were broken on arrival or not sufficiently filled. Total DNA was extracted from 4 mL of plasma by means of the QIAamp DSP Circulating Nucleic Acid Kit (Qiagen, Courtaboeuf, France) after thawing and centrifugation of the samples, then eluted in 55 µL of elution buffer, in accordance with manufacturer’s instructions. The DNA libraries were then prepared in semi-automated 96-microplate format with no size selection, starting from 50 µL of extracted DNA solution using the NEBNext Ultra DNA Library Prep Kit (NEB, Evry, France). After quantification on the LabChip GX microfluidic platform (Perkin-Elmer, Courtaboeuf, France), the libraries from 12 different samples were pooled and sequenced on each lane of an Illumina V3 flow-cell on a HiSeq. 1500 instrument, using the TruSeq SBS kit V3-HS reagent (Illumina, Paris, France) for 27 cycles, followed by 8 cycles to read each sample index.

### Virus genomes

HCMV genome reference (Human herpesvirus 5 strain Merlin, NC_006273) was collected from the NCBI database. Alignment of 58 negative control samples on this reference was performed to identify genomic regions that allow non-specific alignments. We excluded regions with a low complexity (estimated with Shannon entropy) and an abnormally high level of mapped reads. These excluded regions represent 1.01% (2509 bp on 235647 bp) of the total HCMV genome which increases the theoretical specificity without altering the detection sensitivity. To check the alignment quality, we computed the standard deviation of a viral genome depth based on the number of reads aligned at each viral genome position (depth_i_) computed with BEDtools^39^, the mean depth observed on a viral genome (depth_mean_), and length of the viral genome. A threshold, obtained from the negative controls after genome masking, was then used to validate the regular alignment on viral genome for each sample.

We used the same methodology for all viral genome references. All reference genomes are summarized in Supplemental Table 2.

### Bioinformatics analysis

Sequenced reads were filtered to keep only high-quality reads with a standard criterion: all reads with less than 15 consecutive bases with quality greater than or equal to 25 were removed. The total number of reads with sufficient quality was checked to identify samples with insufficient sequenced reads. Only samples with more than 10 million sequenced reads were retained for further analysis.

A first step of alignment on human genome (GRCh38) was performed using Bowtie^40^. To efficiently filter the human reads, we allowed no mismatches and did not guarantee the best alignment location. We performed an additional quality check and discarded any sample with less than 65% of reads aligned on the human genome, leading to the removal of 8 samples.

For virus identification, we aligned the remaining reads to the viral genome using Bowtie. To improve the alignment quality, we allowed the alignment of reads with a unique location on the genome and authorized two mismatches between reads and the viral genome. We also removed duplicate reads using Picard tools (http://broadinstitute.github.io/picard/).

After mapping to the virus reference genome, we normalized the number of viral reads with the total number of sequenced reads for each sample and computed mean depth. The mean depth (depth_mean_) depends on the normalized number of reads aligned to the viral genome (N), the length of this genome (L) and the size of the sequenced reads (size_reads_). The mean depth value, computed as described in Equation (1), directly represents the quantity of reads aligned to an exogenous genome independently of its length that can influence the quantity of exogenous reads present in a biologic sample.

1 $$dept{h}_{mean}=\frac{N\,x\,siz{e}_{reads}}{L}$$

We finally constructed a linear regression model based on HCMV calibration samples to estimate the viral load of unknown samples. The regression model was computed using the linear model function (lm) from R with default parameters.

All data processing and analysis from reads obtained from sequencer to the final report with the viral load estimation was coded using snakemake^41^, a workflow engine which calls R (https://www.r-project.org/) and python (https://www.python.org/) scripts and shell (bash) command lines.

## Electronic supplementary material

Supplementary Information
