# Supplementary material for: Using massively parallel shotgun sequencing of maternal plasmatic cell-free DNA for cytomegalovirus DNA detection during pregnancy: a proof of concept study
Source: Sci Rep. 2018 Mar 12;8:4321. doi: 10.1038/s41598-018-22414-6 (PMC5847603; doi:10.1038/s41598-018-22414-6)
Supplement: Supplementary file 1 — Supplementary Information [file 41598_2018_22414_MOESM1_ESM.pdf]

SUPPLEMENTAL DATA

Using massively parallel shotgun sequencing of maternal plasmatic cell-free DNA for cytomegalovirus DNA detection during pregnancy: a proof of concept study

Virginie CHESNAIS[1*+], Alban OTT[1+], Emmanuel CHAPLAIS[1], Samuel GABILLARD[1], Diego PALLARES[1], Christelle VAULOUP FELLOUS[2], Alexandra BENACHI[3], Jean-Marc COSTA[4], and Eric GINOUX[1*]

[1] Life&Soft, Plessis-Robinson, France

[2] AP-HP, Hôpital Paul Brousse, Groupe Hospitalier Universitaire Paris-Sud, Virologie, Université Paris-Sud, INSERM U1193, Villejuif, France

[3] AP-HP, Hôpital Antoine Béclère, Service de Gynécologie-Obstétrique et Médecine de la Reproduction, Université Paris-Sud, Clamart, France

[4] Laboratoire CERBA, Saint-Ouen-l'Aumône, France

[*] vchesnais@lifeandsoft.com, eginoux@lifeandsoft.com

[+] these authors contributed equally to this work

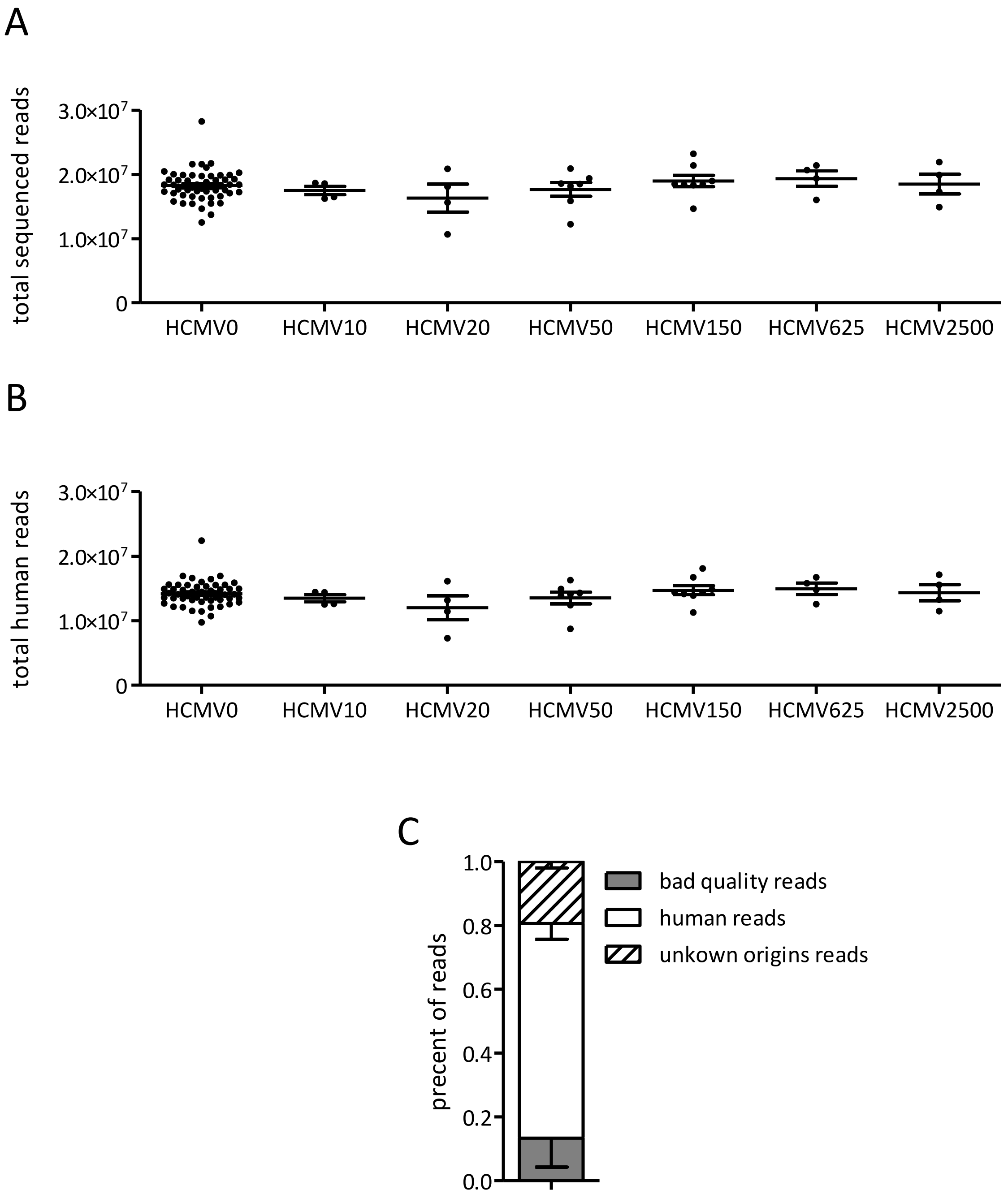

Supplemental figure 1 : Sequencing results for HCMV calibration samples. (A) Summary of raw sequencing results of the HCMV calibration samples. The total number of sequenced reads was represented. Data are shown as Tukey boxplots. The bottom and top of the box indicates the upper and lower quartiles and the band inside the box indicates the median. (B) Summary of raw sequencing results of the 538 plasma samples. The total number of human

sequenced reads were represented. Data are shown as Tukey boxplots. The bottom and top of the box indicates the upper and lower quartile and the band inside the box indicates the median. (C) Proportion of bad quality, human and unkown origin reads for all HCMV calibration samples. The Standard Error of the Mean (SEM) is represented for each proportion.

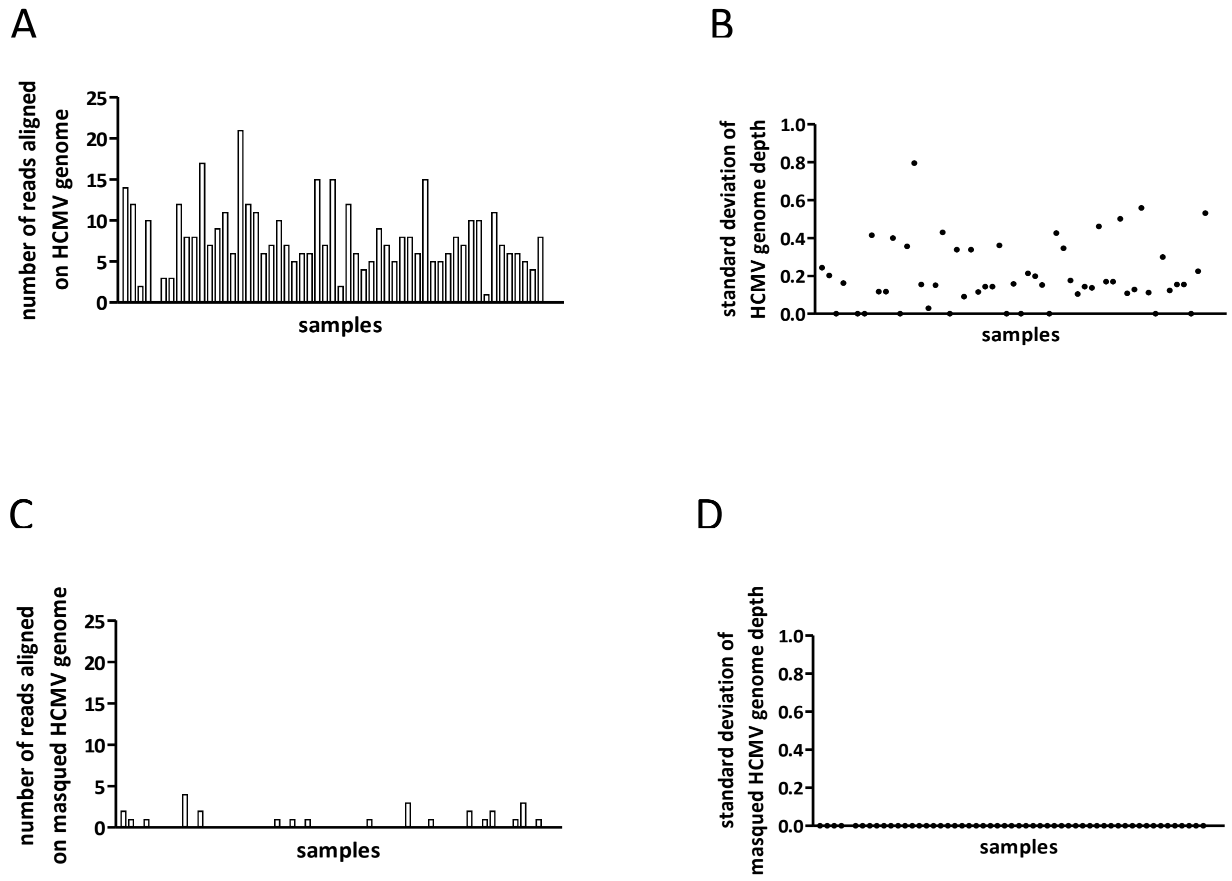

Supplemental figure 2 : Summary of mapping to the HCMV and masked HCMV genome. (A) Representation of the number of reads aligned to the HCMV genome. Each bar represents a result obtained for one HCMV negative sample. (B) Representation of the standard deviation of depth after alignment of reads to the HCMV genome. Each point represents a result obtained for one HCMV negative sample. (C) Representation of the number of reads aligned to the HCMV masked genome. Each bar represents a result obtained for one HCMV negative sample. (D) Representation of the standard deviation of depth after alignment of reads to the HCMV masked genome. Each point represents a result obtained for one HCMV negative sample.

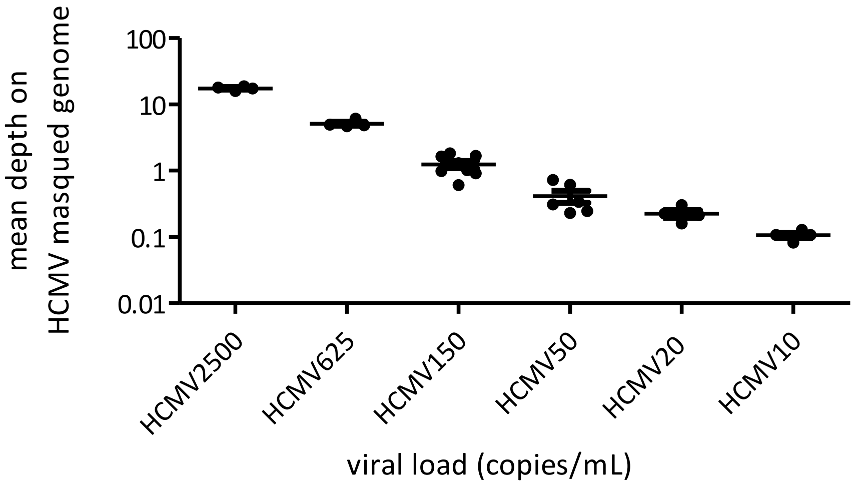

Supplemental figure 3 : Alignment results of ranged HCMV samples. Mean depth obtained on the HCMV masked genome for the 6 groups of HCMV calibration samples with a viral load from 2500 copies/mL to 10 copies/mL.

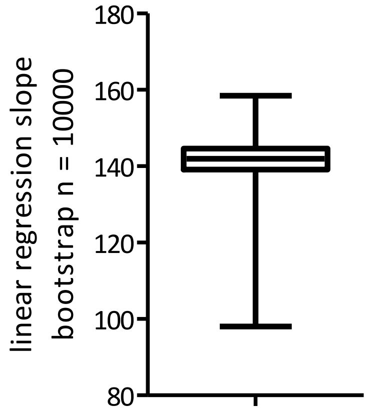

Supplemental figure 4 : Linear regression slopes obtained from bootstrap analysis. Box plot representation of all linear regression slopes obtained after bootstrap tests (n=10000) based on all HCMV calibration samples.

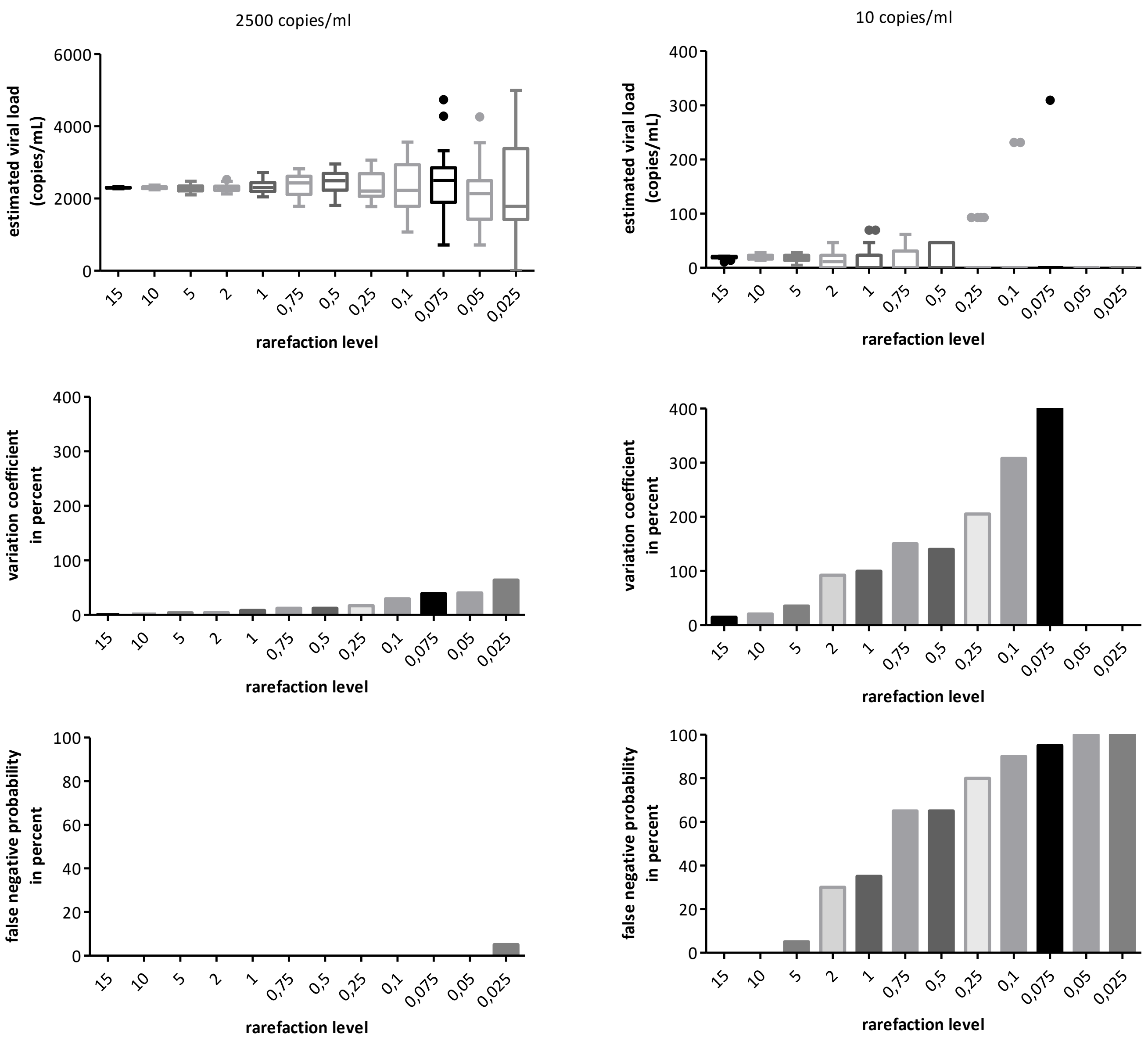

Supplemental figure 5 : Callibration samples rarefaction results. Rarefaction analysis was performed for 2 calibration samples with a viral load of respectively 2500 copies/ml and 10 copies/ml. For each rarefaction levels (expressed in million reads), 20 draws were aleatory made from a single fastq file. The estimated viral load, the variation coefficient of the 20 estimated viral load and the probability to detect a fasle positive were represented for each draw with decreased simulated depth.

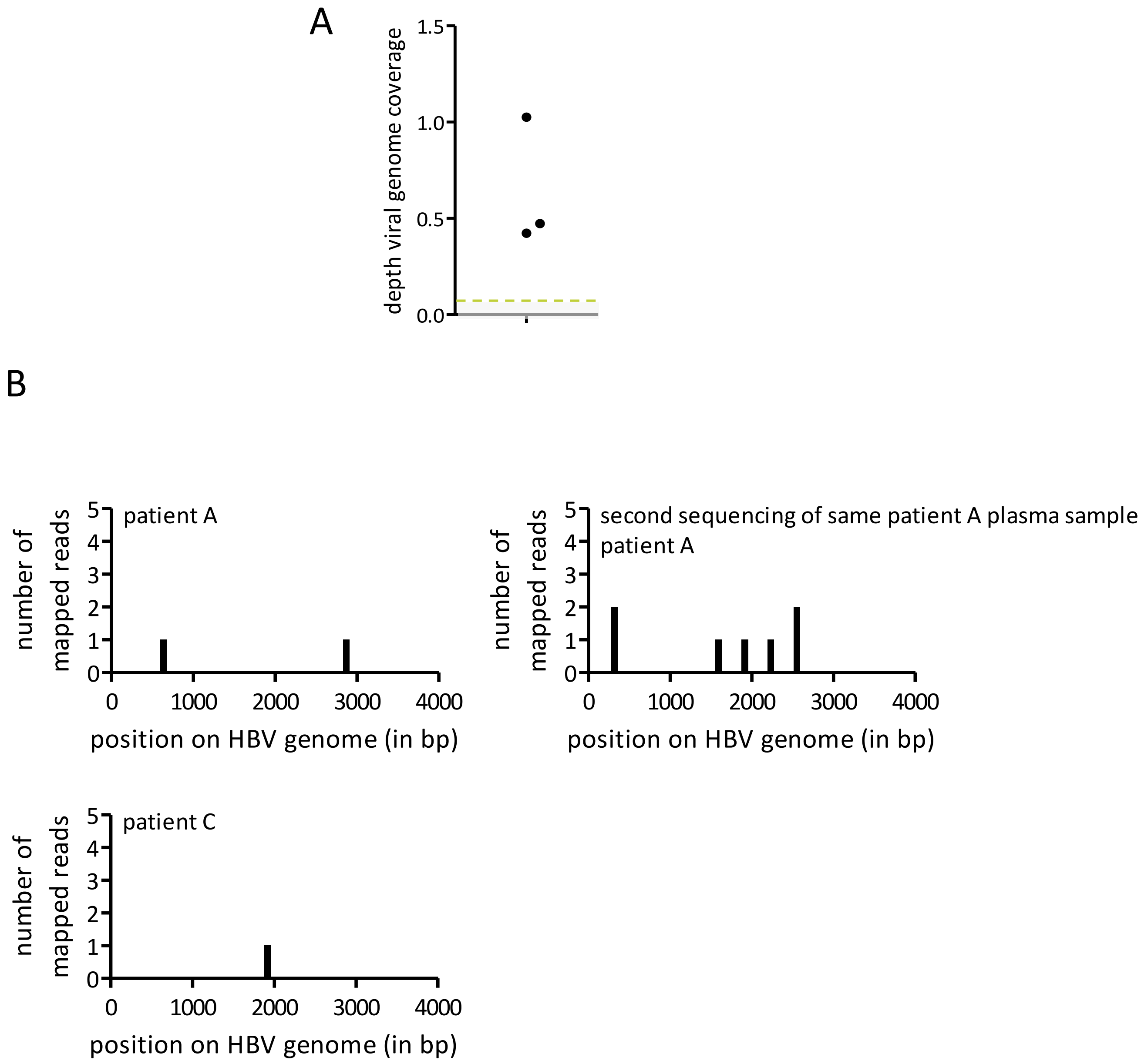

Supplemental figure 6 : HBV sequencing results of the retrospective pregnant patient cohort. (A) Mean depth on masked HCMV samples for the 538 plasma samples were represented. The grey zone represents samples with a mean depth lower than the positive threshold for detection of HBV circulating DNA. (B) Detailed alignment of reads for positive samples. For each plot, the number of reads aligned at all HBV genome positions (in bp) is represented. The results are shown for the sequencing of three patients: patient A and patient C.

Sample Name	HCVM2500	HCVM625	HCVM150	HCVM50	HCVM20	HCVM10	HCMV0
**Viral load (copies/mL)**	2500	625	150	50	20	10	0
**Number of samples**	N=4	N=4	N=8	N=7	N=4	N=4	N=58

Supplemental table 1 : Description of the HCVM calibration samples.

Reference	Description
NC_006273.2	Human herpesvirus 5 strain Merlin, complete genome
NC_001798.2	Human herpesvirus 2 strain HG52, complete genome
NC_001348.1	Human herpesvirus 3, complete genome
NC_003977.2	Hepatitis B virus (strain ayw) genome
NC_004500.1	Human papillomavirus type 92, complete genome
NC_005134.2	Human papillomavirus type 96, complete genome
NC_017993.1	Human papillomavirus type 135, complete genome
NC_017994.1	Human papillomavirus type 136, complete genome
NC_017995.1	Human papillomavirus type 137, complete genome
NC_017996.1	Human papillomavirus type 140, complete genome
NC_017997.1	Human papillomavirus type 144, complete genome
NC_012213.1	Human papillomavirus type 108, complete genome
NC_021483.1	Human papillomavirus type 154 isolate PV77, complete genome
NC_001690.1	Human papillomavirus type 48, complete genome
NC_001691.1	Human papillomavirus type 50, complete genome
NC_001354.1	Human papillomavirus type 41, complete genome
NC_001457.1	Human papillomavirus type 4, complete genome
NC_001458.1	Human papillomavirus type 63, complete genome
NC_001576.1	Human papillomavirus type 10, complete genome
NC_001583.1	Human papillomavirus type 26, complete genome
NC_001586.1	Human papillomavirus type 32, complete genome
NC_001595.1	Human papillomavirus type 7, complete genome
NC_001596.1	Human papillomavirus type 9, complete genome
NC_001591.1	Human papillomavirus type 49, complete genome
NC_001587.1	Human papillomavirus type 34, complete genome
NC_016157.1	Human papillomavirus type 126, complete genome

NC_010329.1	Human papillomavirus type 88, complete genome
NC_008188.1	Human papillomavirus type 103, complete genome
NC_001694.1	Human papillomavirus - 61, complete genome
NC_001693.1	Human papillomavirus type 60, complete genome
NC_001676.1	Human papillomavirus 54, complete genome
NC_001531.1	Human papillomavirus - 5, complete genome
NC_012486.1	Human papillomavirus type 112, complete genome
NC_012485.1	Human papillomavirus type 109, complete genome
NC_013035.1	Human papillomavirus 116, complete genome
NC_014956.1	Human papillomavirus type 134, complete genome
NC_008189.1	Human papillomavirus type 101, complete genome
NC_004104.1	Human papillomavirus type 90, complete genome
NC_001352.1	Human papillomavirus - 2, complete genome
NC_001355.1	Human papillomavirus type 6b, complete genome
NC_014185.1	Human papillomavirus 121, complete genome
NC_014952.1	Human papillomavirus type 128, complete genome
NC_014953.1	Human papillomavirus type 129, complete genome
NC_014954.1	Human papillomavirus type 131, complete genome
NC_014955.1	Human papillomavirus type 132, complete genome
NC_023891.1	Human papillomavirus type 178, complete genome
NC_019023.1	Human papillomavirus type 166 isolate KC9, complete genome
NC_001356.1	Human papillomavirus - 1, complete genome
NC_001526.2	Human papillomavirus type 16, complete genome
NC_028125.1	Human papillomavirus type 163 isolate KC3, complete genome
NC_026946.1	Human papillomavirus KC5, complete genome
NC_001593.1	Human papillomavirus type 53, complete genome
NC_001357.1	Human papillomavirus - 18, complete genome
NC_000883.2	Human parvovirus B19, complete genome

NC_009333.1	Human herpesvirus 8, complete genome
NC_009334.1	Human herpesvirus 4, complete genome
NC_000898.1	Human herpesvirus 6B, complete genome
NC_001716.2	Human herpesvirus 7, complete genome
NC_001664.2	Human herpesvirus 6A, complete genome
NC_001806.2	Human herpesvirus 1 strain 17, complete genome

Supplemental table 2 : List of the viral reference genomes used in the study.
